# Time-restricted ketogenic diet in amyotrophic lateral sclerosis: a case study

**DOI:** 10.3389/fneur.2023.1329541

**Published:** 2024-01-18

**Authors:** Matthew C. L. Phillips, Samuel E. Johnston, Pat Simpson, David K. Chang, Danielle Mather, Rognvald J. Dick

**Affiliations:** ^1^Department of Neurology, Waikato Hospital, Hamilton, New Zealand; ^2^Older Persons and Rehabilitation Service, Waikato Hospital, Hamilton, New Zealand; ^3^Department of Respiratory Medicine, Waikato Hospital, Hamilton, New Zealand; ^4^Department of Speech Language Therapy, Waikato Hospital, Hamilton, New Zealand

**Keywords:** motor neuron disease, amyotrophic lateral sclerosis, neurodegeneration, energy metabolism, mitochondria dysfunction, metabolic strategy, fasting, ketogenic diet

## Abstract

Amyotrophic lateral sclerosis (ALS) is an incurable neurodegenerative disorder. The most devastating variant is bulbar-onset ALS, which portends a median survival of 24 months from the onset of symptoms. Abundant evidence indicates that neuron metabolism and mitochondrial function are impaired in ALS. Metabolic strategies, particularly fasting and ketogenic diet protocols, alter neuron metabolism and mitochondria function in a manner that may mitigate the symptoms of this disorder. We report the case of a 64-year-old man with a 21-month history of progressive, deteriorating bulbar-onset ALS, with an associated pseudobulbar affect, who implemented a time-restricted ketogenic diet (TRKD) for 18 months. During this time, he improved in ALS-related function (7% improvement from baseline), forced expiratory volume (17% improvement), forced vital capacity (13% improvement), depression (normalized), stress levels (normalized), and quality of life (19% improvement), particularly fatigue (23% improvement). His swallowing impairment and neurocognitive status remained stable. Declines were measured in physical function, maximal inspiratory pressure, and maximal expiratory pressure. Weight loss was attenuated and no significant adverse effects occurred. This case study represents the first documented occurrence of a patient with ALS managed with either a fasting or ketogenic diet protocol, co-administered as a TRKD. We measured improved or stabilized ALS-related function, forced expiratory volume, forced vital capacity, swallowing, neurocognitive status, mood, and quality of life. Measurable declines were restricted to physical function, maximal inspiratory pressure, and maximal expiratory pressure. Now over 45 months since symptom onset, our patient remains functionally independent and dedicated to his TRKD.

## Introduction

Amyotrophic lateral sclerosis (ALS) is an incurable neurodegenerative disorder that afflicts 4.1–8.4 out of every 100,000 people in the world (1), with the number of new cases anticipated to rise over the next 20–25 years (2). Although most patients develop limb-onset ALS, 25–30% of patients develop the more devastating bulbar-onset variant, which is associated with a median survival of 24 months from the onset of symptoms (3). Bulbar-onset ALS typically presents with dysarthria, dysphagia, or dysphonia. Many patients also develop changes in behavior, cognition, and mood. The pathogenesis of ALS involves the degeneration and death of upper motor neurons in the brain motor cortices, lower motor neurons in the brainstem and spinal cord, and neurons in the frontotemporal regions of the brain (4). Approximately 90% of patients lack a family history and are classified as sporadic ALS, whereas the remaining 10% show a pattern of inheritance associated with a gene mutation and are classified as familial. Treatment for sporadic and familial ALS is limited and new therapies are needed.

Abundant evidence indicates that neuron metabolism and mitochondrial function are impaired in ALS (5–10). On a morphological level, mitochondria are abnormally shaped, swollen, and vacuolated (6, 11). Metabolically, human-derived motor neurons in both sporadic and familial ALS exhibit defective oxidative phosphorylation, ATP loss, and elevated levels of reactive oxygen species (9, 12). Mitochondria in ALS motor neurons also display impaired glucose metabolism, tricarboxylic acid (TCA) cycle activity, calcium buffering, axonal transport, and population dynamics (5, 10). Moreover, degradations in cell metabolism and mitochondria function have been documented in astrocytes, microglia, Schwann cells, hepatocytes, lymphocytes, and skeletal muscle cells (6). Collectively, these impairments in cell metabolism and mitochondria function culminate in a chronic bioenergetic challenge that disproportionately impacts metabolically active cells such as neurons, glia, and myocytes.

Metabolic strategies, particularly fasting and ketogenic diet protocols, alter cell metabolism and mitochondria function (13). Both strategies enhance production of the dominant blood ketone, beta-hydroxybutyrate (BHB), such that its concentration is sustained at 0.5–0.6 mmol/L or higher (14). BHB metabolism leads to an enhanced free energy of ATP hydrolysis and a greater supply of TCA cycle intermediates (15). Ketone metabolism also produces fewer reactive oxygen species and increases the production of oxidative stress resistance factors (16, 17). Importantly, fasting and ketogenic diet regimens renew the mitochondria pool by upregulating mitogenesis and mitophagy (18). Despite these beneficial effects, current clinical evidence for metabolic strategies in ALS is limited to a handful of studies involving transgenic mouse models—for example, compared with mice maintained on a normal diet, mice sustained on a ketogenic diet show preserved motor performance (19), and mice fed caprylic triglyceride (a medium-chain fatty acid that is readily metabolized into ketones) show a delayed progression of weakness, improved performance, and protection from motor neuron loss (20).

Given the collective evidence, we hypothesized that a metabolic strategy might lead to clinical benefits and improved quality of life in a patient with ALS.

## Case study

We report the case of a 64-year-old male dairy farm systems stock controller of European background who presented to his general practitioner with 9 months of slurring and slowing of speech, difficulty swallowing solids, coughing when consuming liquids, intermittent sialorrhea, and constant fatigue. His family had also noted intermittent episodes of laughing and sobbing. He was referred to our motor neuron disease clinic, which occurred 9 months later due to resource constraints, by which time his bulbar symptoms had worsened leading to 10 kg of weight loss since symptom onset. Medical history included colorectal cancer (T1N0M0) 12 years previously, which was treated with a hemicolectomy, as well as a myocardial infarction 11 years previously, treated with coronary angioplasty. He had a 2-year history of chronic musculoskeletal pain afflicting both shoulders, hips, and ankles. Regular medications included aspirin and metoprolol. He was a life-long smoker (10 cigarettes a day for 50 years—he had quit 4 years previously, but recently resumed smoking due to frustration over his symptoms). There was no family history of neuromuscular disease. Socially, he lived with his wife, who was also his caregiver, and had recently retired from work due to his symptoms. On examination, our patient had a muscular build, with little body fat, and he exhibited inappropriate laughter and crying throughout the consultation. He was 160 cm in height and 72.2 kg in weight, with a calculated body-mass index of 28.2 kg/m^2^. Neurological examination of the bulbar region revealed definite tongue wasting and fasciculations, a brisk jaw jerk, and spastic dysarthria. Examination of the cervical region revealed mild wasting of the bilateral supraspinatus and infraspinatus muscles and mild weakness in left shoulder abduction, elbow flexion and extension, and finger grip and abduction (all 5-/5). All limb reflexes were normal (2+) and plantar responses were normal. Electromyography of the bulbar, cervical, thoracic, and lumbosacral regions revealed moderate to severe chronic denervation (reduced recruitment and large-amplitude, long-duration motor unit action potentials) in multiple muscles in all four regions. MRI brain showed mild diffuse leukoariosis and MRI spine showed multilevel bilateral foraminal stenoses in the cervical spine, both considered normal for age by a neuroradiologist. An ALS panel analysis for mutations in 35 different genes was negative (ALS Panel, Blueprint Genetics, Espoo, Finland). Blood investigations were normal. At the 3-month follow-up, 21 months after symptom onset, our patient was diagnosed with bulbar-onset ALS with an associated pseudobulbar affect by two independent neurologists (both with neurophysiology fellowships) based on the 2020 Gold Coast criteria (21).

**Figure 1:**
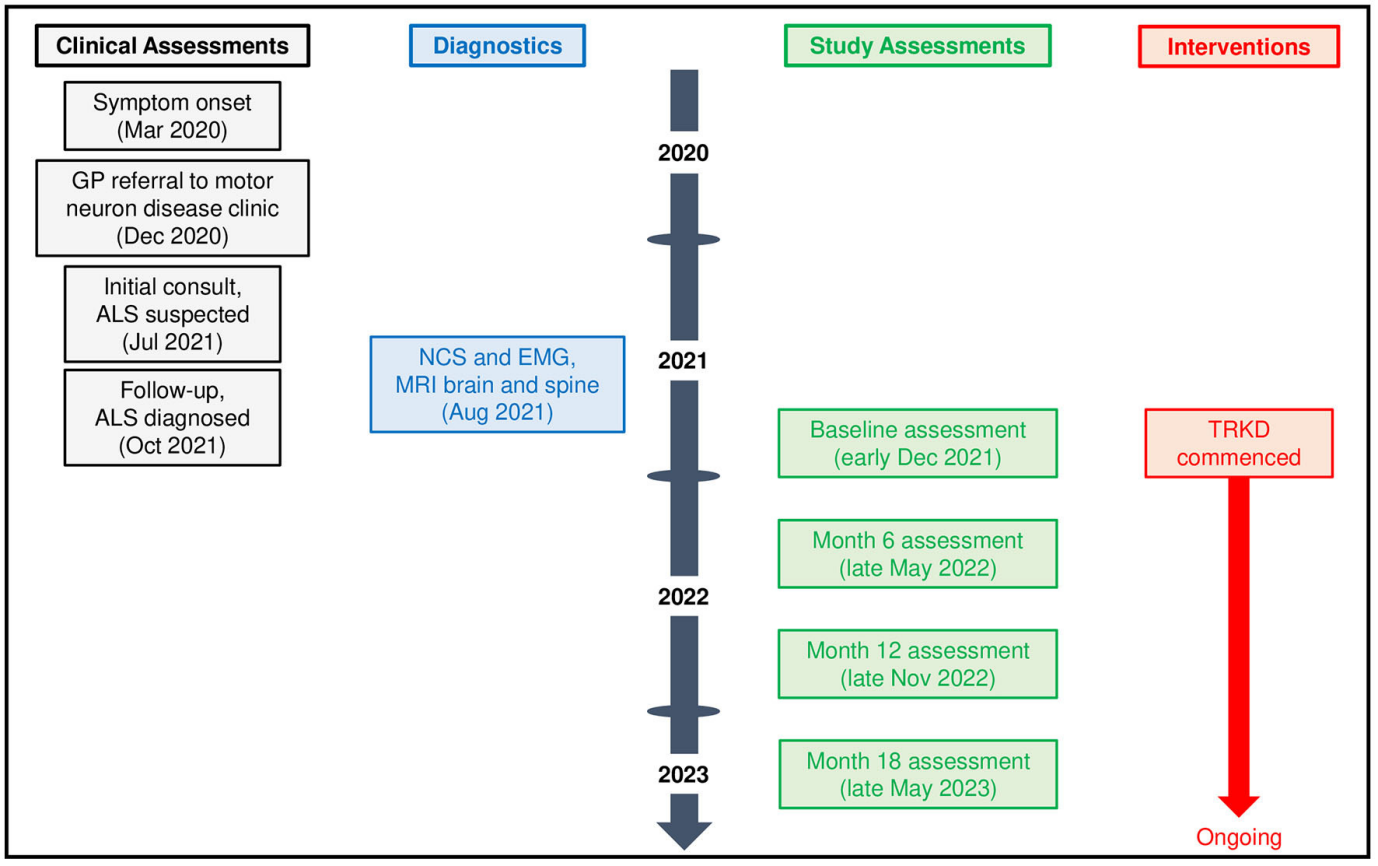
Patient timeline.

Given the deteriorating symptoms, riluzole was offered but our patient declined, after which an 18-month time-restricted ketogenic diet (TRKD) was presented as an option (Figure 1). The TRKD involved reducing feeding times to two meals a day. Our patient chose the timing of the two meals every day and up to 1 h was permitted per meal, ensuring that food intake was limited to 2 h per day, with fasting (allowing only water, tea, and coffee) occurring all other hours. The modified ketogenic diet was roughly 60% fat, 30% protein, 5% fiber, and 5% net carbohydrate by weight and comprised primarily of whole foods (green vegetables, meats, eggs, nuts, seeds, creams, and natural oils). Our patient was encouraged to eat to satiation at every meal and not to restrict his calorie intake. After obtaining written informed consent, we provided him with a booklet containing guidelines, recipes, and space to record daily (bedtime) blood glucose and ketone levels using a blood glucose and ketone monitor (CareSens Dual, Pharmaco Diabetes, Auckland, New Zealand). The lead investigator provided support as needed via email. Aside from the TRKD, there were no other lifestyle changes and our patient continued to smoke 10 cigarettes a day.

Clinical assessments were conducted during the week preceding the start of the TRKD, with repeat assessments at 6, 12, and 18 months. Clinical assessments evaluated ALS-related function, physical function, pulmonary function, swallowing impairment, neurocognitive status, mood, and quality of life. Assessors were blinded to the intervention. ALS-related function was reported by the patient and caregiver using the Revised ALS Functional Rating Scale (ALSFRS-R) (scores range from 0 to 48, higher numbers indicate improved function) (22). Physical function was measured by a physiotherapist using the get-up-and-go, 6-min walk, and stair climb tests, with an average from two tests calculated for each measure (23–25). Pulmonary function was measured by a pulmonary clinical nurse specialist using spirometry, which assessed forced expiratory volume, forced vital capacity, maximal inspiratory pressure, and maximal expiratory pressure. Swallowing impairment was assessed by speech and language therapists with videofluoroscopic swallowing study and interpreted using the New Zealand Index for Multidisciplinary Evaluation of Swallowing (NZIMES, available at https://fliphtml5.com/nfqi/zyar/basic), which delineates the oral phase, oral transit parameters, pharyngeal phase, crico-esophageal parameters, and laryngeal parameters. Neurocognitive status was measured by a neuropsychologist using the Repeatable Battery for the Assessment of Neuropsychological Status (RBANS, with Form A used at baseline and Week 12 and Form B used at Weeks 6 and 18) (26), Processing Speed from the Wechsler Adult Intelligence Scale—Fourth Edition (27), the Trail Making Test from the Delis-Kaplan Executive Function System (28), and the Controlled Oral Word Association Test (COWAT) from the Multilingual Aphasia Examination (29) (for all tests, higher numbers indicated improved neurocognition), as well as mood using the Depression Anxiety Stress Scale (30). Quality of life was reported by the patient and caregiver using the Functional Assessment of Chronic Illness Therapy—Fatigue (FACIT-F) (scores range from 0 to 160, higher numbers indicate better quality of life) (31). Body weight and blood markers were measured at each assessment. We conducted an adverse effects questionnaire with the patient and caregiver at 6, 12, and 18 months.

**Figure 2:**
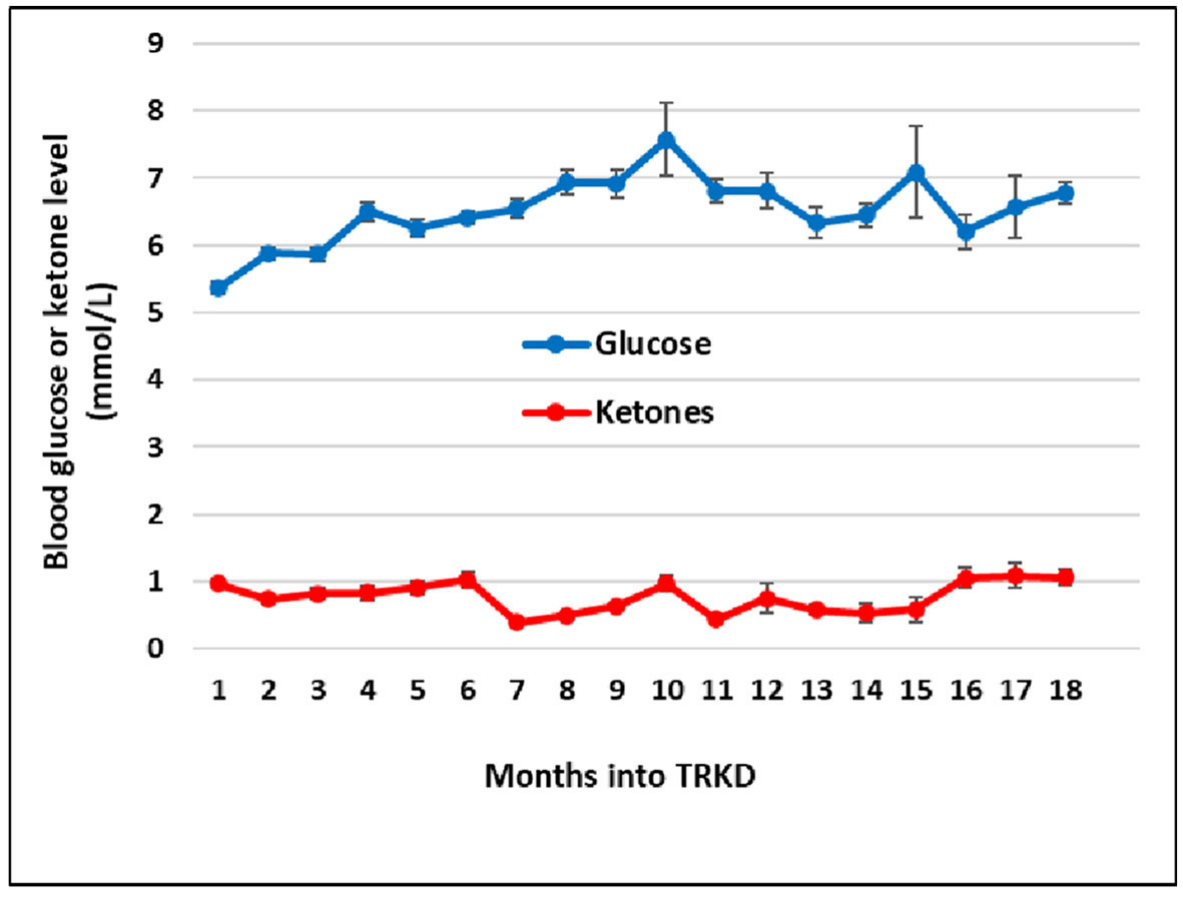
Mean monthly blood glucose and ketone (beta-hydroxybutyrate) levels during the time-restricted ketogenic diet (TRKD). Error bars indicate standard error.

During the 18-month TRKD, our patient's mean blood glucose and ketone levels were 6.52 +/- 0.91 and 0.77 +/- 0.43 mmol/L, respectively (Figure 2), with improvement or stability documented in most outcome measures (Tables 1, 2). Regarding function, the ALSFRS-R improved (42–45, representing a 7% improvement from baseline). The get-up-and-go, 6-min walk, and stair climb tests showed decline. Regarding pulmonary function, the forced expiratory volume improved (2.31–2.70 L, representing a 17% improvement), as did forced vital capacity (3.83–4.33 L, representing a 13% improvement). The maximal inspiratory and expiratory pressures declined. Baseline swallowing impairment remained stable aside from the oral phase (none to mild impairment) and laryngeal parameters (moderate to mild impairment). Despite his stabilized swallowing function, our patient opted for a radiologically inserted gastrostomy (RIG) insertion 9 months into the TRKD to mitigate symptoms of aspiration, which existed before the TRKD was implemented and were only triggered by water (to date, the RIG remains solely used for water intake). The neurocognitive tests generally remained stable. Baseline depression and stress levels resolved (moderate to normal). Regarding quality of life, the FACIT-F improved (114–136, representing a 19% improvement), particularly fatigue as measured by the FACIT-F subscale (35–43, representing a 23% improvement). Our patient's weight was 72.2 kg at baseline and 69.0 kg at month 18. Blood investigations for hemoglobin, creatinine, liver function tests, and glycosylated hemoglobin remained normal over 18 months. Blood triglycerides increased from 1.3 to 1.6 mmol/L, high-density lipoprotein remained at 1.1 mmol/L, low-density lipoprotein increased from 3.1 to 5.4 mmol/L, and total cholesterol increased from 4.8 to 7.2 mmol/L. Our patient experienced no significant adverse effects during the TRKD and consistently mentioned enhanced energy, improved sleep, and reductions in his chronic musculoskeletal pain.

**Table 1:** Outcome measures for ALS-related, physical, pulmonary, and swallowing function at baseline, 6 months, 12 months, and 18 months after commencing the time-restricted ketogenic diet (for all tests except the get-up-and-go and stair climb, higher numbers indicate improved outcomes; improved or stabilized outcomes are highlighted in blue and declines are highlighted in red).

**Outcome**	**Baseline**	**Month 6**	**Month 12**	**Month 18**
**ALS-related function**				
ALSFRS-R				
Speech	3	3	3	3
Salivation	2	2	2	3
Swallowing	3	3	3	3
Handwriting	4	4	3	4
Handling utensils	4	4	4	4
Dressing and hygiene	4	4	4	4
Turning in bed	4	4	4	4
Walking	4	4	4	4
Climbing stairs	3	4	4	4
Dyspnea	3	4	4	4
Orthopnea	4	4	4	4
Respiratory insufficiency	4	4	4	4
Total	42	44	43	45
**Physical function**				
Get-up-and-go (s)	6.9	6.6	6.8	7.9
6-min walk (m)	541	568	538	497
Stair climb (s)	8.9	8.3	9.3	10.5
**Pulmonary function**				
FEV1 (L, % predicted)	2.31, 86	2.62, 97	2.37, 88	2.70, 99
FVC (L, % predicted)	3.83, 111	3.87, 112	4.14, 120	4.33, 124
MIP (kPa, % predicted)	5.86, 70	4.10, 50	5.00, 60	4.10, 50
MEP (kPa, % predicted)	11.30, 86	12.20, 93	6.70, 51	4.10, 31
**Swallowing impairment**				
Oral phase	None	Mild	Mild	Mild
Oral transit parameters	None	Mild	Mild	None
Pharyngeal phase	Mild	Mild	Mild	Mild
Crico-esophageal parameters	None	None	None	None
Laryngeal parameters	Moderate	None	None	Mild

ALSFRS-R, Revised Amyotrophic Lateral Sclerosis Functional Rating Scale; FEV1, Forced Expiratory Volume; FVC, Forced Vital Capacity; MIP, Maximal Inspiratory Pressure; MEP, Maximal Expiratory Pressure.

**Table 2:** Outcome measures for neurocognitive status, mood, and quality of life at baseline, 6 months, 12 months, and 18 months after commencing the time-restricted ketogenic diet (for all tests, higher numbers indicate improved outcomes; improved or stabilized outcomes are highlighted in blue and declines are highlighted in red).

**Outcome**	**Baseline**	**Month 6**	**Month 12**	**Month 18**
**Neurocognitive status**				
RBANS (scaled score, percentile)				
Immediate memory	76, 5	76, 5	81, 10	85, 16
Visuospatial/constructional	100, 50	100, 50	105, 63	131, 98
Language	96, 39	96, 39	92, 30	92, 30
Attention	91, 27	82, 12	91, 27	85, 16
Delayed memory	98, 45	100, 50	102, 55	100, 50
Total	88, 21	86, 18	91, 27	97, 42
Processing Speed (scaled score, percentile)	97, 42	86, 18	100, 50	94, 34
Trail Making Test (scaled score, percentile)				
Visual scanning	12, 75	7, 16	10, 50	10, 50
Number sequencing	14, 91	12, 75	13, 84	11, 63
Letter sequencing	12, 75	12, 75	13, 84	12, 75
Number switching	11, 63	11, 63	10, 50	11, 63
Motor speed	11, 63	10, 50	11, 63	11, 63
COWAT (raw score, percentile)				
Phonological fluency	18, < 10	18, < 10	18, < 10	17, < 10
Semantic fluency	22, 90	21, 90	18, 75	19, 75
**Mood**				
Depression Anxiety Stress Scale				
Depression	Moderate	Moderate	Normal	Normal
Anxiety	Normal	Moderate	Normal	Normal
Stress	Moderate	Moderate	Normal	Normal
**Quality of life**				
FACIT-F (overall)	114	135	141	136
FACIT-F subscale (fatigue)	35	43	42	43

RBANS, Repeatable Battery for the Assessment of Neuropsychological Status; COWAT, Controlled Oral Word Association Test; FACIT-F, Functional Assessment of Chronic Illness Therapy - Fatigue.

## Discussion

This case study represents the first documented occurrence of a patient with ALS managed with either a fasting or ketogenic diet protocol, co-administered as a TRKD. We measured improved ALS-related function (7% improvement from baseline), forced expiratory volume (17% improvement), forced vital capacity (13% improvement), depression (normalized), stress levels (normalized), and quality of life (19% improvement), particularly fatigue (23% improvement). His swallowing impairment and neurocognitive status remained stable. Measurable declines were restricted to physical function, maximal inspiratory pressure, and maximal expiratory pressure. Weight loss was attenuated and no significant adverse effects occurred.

Although fasting and ketogenic diet protocols may individually confer metabolic benefits in ALS, they can be readily combined. Time-restricted feeding eases the burden of organizing multiple meals, which compensates for the extra time required to become familiar with a ketogenic diet, whereas the diet may improve long-term hunger (32), which increases the tolerability of the fasts. The modified ketogenic diet used here was simple, flexible, palatable, and affordable, which alleviated the restrictions that have been associated with ketogenic diets in the past (33). Regardless of whether metabolic strategies are isolated or combined, it is important to monitor blood glucose and BHB levels so that difficulties can be detected and resolved. Despite good adherence to the 18-month TRKD, our patient's mean blood glucose levels averaged 6.52 mmol/L and his BHB 0.77 mmol/L, which is in the lower range of physiological ketosis (albeit, still within range). This may relate to his body composition and the ALS process itself. Our patient's lower fat mass probably provided less fuel reserve for fasting-induced ketogenesis. Moreover, many individuals with ALS exhibit skeletal muscle hypermetabolism (5), which may constitute an adaptive response to decreased energy metabolism efficiency (34). Hypermetabolism could have increased our patient's ketone utilization, leading to lower blood levels.

During this case study, our patient improved or stabilized in most measures of function, which is consistent with TRKD-induced enhancements in neuron, glial cell, and myocyte metabolism as well as mitochondria function. The ALS community relies on the ALSFRS-R to monitor activities of daily living and disease progression (4, 22), which typically declines by 1 point per month (35). Given our patient's baseline score of 42, his score should have declined to 24 during the TRKD. Instead, it improved to 45. Although ALSFRS-R score changes do not necessarily reflect improvement (4), and the specific tests of physical function declined at the final assessment, the fact that our patient's score did not decline over 18 months is potentially important. With respect to pulmonary function, forced vital capacity is arguably the most significant spirometric correlate of disease progression in ALS (36), which shows an average decline of 2–3% predicted per month (35). Given our patient's baseline of 3.83 L (111% predicted), his score should have declined to at least 2.87 L (75% predicted) during the TRKD. Instead, it improved to 4.33 L (124% predicted). Importantly, the maximal inspiratory and expiratory pressures declined during the TRKD, which is discordant with the improved forced expiratory volume and vital capacity, but these measurements should be viewed cautiously as both tests are notoriously difficult to perform and poorly predictive of respiratory capacity (37). Lastly, swallowing impairment occurs in 85–92% of patients diagnosed with bulbar-onset ALS and is associated with malnutrition, aspiration pneumonia, faster functional decline, and increased mortality (38). Importantly, our patient's swallowing function remained stable during the 18-month TRKD, with only minor variations noted in the oral phase (slightly declined) and laryngeal parameters (slightly improved), both of which can be attributed to typical variations seen from swallow to swallow.

Our patient improved or remained stable in most measures of neurocognitive status, mood, and quality of life. Although ALS is often considered a neuromuscular disorder, 50% of patients exhibit impaired executive function, language fluency, or cognition due to pathology in the frontotemporal region (39, 40). The RBANS measures a variety of cognitive domains related to memory, visuospatial and constructional ability, expressive language, and attention (26). Our patient's low baseline RBANS scores (21st percentile) suggested a degree of baseline executive-type impairment, which remained stable (or improved) after the 18-month TRKD (42nd percentile). The COWAT measures verbal fluency (29). Our patient's low baseline phonological fluency scores (< 10th percentile) indicated substantial language impairment at baseline, which remained stable. The ongoing stability in most of the neurocognitive tests is encouraging and may reflect TRKD-associated enhancements in neocortical neuron metabolism and mitochondria function. From the perspective of our patient and his wife, the greatest benefits associated with the TRKD related to his mood and energy levels. His moderate baseline depression and stress levels resolved. He also improved his quality of life and fatigue scores, which allowed him to maintain an active outdoors lifestyle on his farm.

During the 21 months prior to the TRKD, our patient lost 10 kg of body weight, which was concerning given that weight loss is negatively correlated with survival in ALS (7, 8). By contrast, he lost only 3.2 kg of weight during the 18-month TRKD. Although the resumption of his smoking habit may have accounted for a portion of the 10 kg of weight loss, the relative weight preservation during the TRKD hints at a weight-sparing effect. It might seem paradoxical that a strategy with the potential to induce a caloric deficit could somehow lead to a weight-sparing effect in ALS. However, the explanation may lie in a TRKD-induced increased efficiency of ATP synthesis. ALS involves the accumulation of damaged, uncoupled mitochondria (41), followed by a progressive decrease in energy metabolism efficiency, energy dissipation through thermogenesis, and ATP depletion despite failing attempts by hypermetabolism to compensate for the shortfall. Fasting may counter this process by inducing mitochondria renewal and coupling, which can increase the rate of ATP synthesis—for example, in a study involving uncoupling protein-3 knockout mice, fasted mice showed a four-fold higher rate of ATP synthesis compared to fed mice despite no measurable difference in TCA cycle activity or whole-body energy expenditure (42). The TRKD may have increased ATP synthesis efficiency in our patient, culminating in less generated energy being “lost” and a subsequent sparing effect on muscle and fat reserves. Consistent with this hypothesis, well-designed trials have shown that simply increasing calorie intake, which would not increase metabolic efficiency, does not significantly attenuate weight loss in people with ALS (43, 44).

Given that this is a case study, we cannot draw firm conclusions regarding the mechanism of the documented improvements or potential impact of the TRKD on survival. Clinical features associated with shorter survival times include bulbar-onset ALS, older age, rapid functional decline (as measured by the ALSFRS-R), low forced vital capacity, frontotemporal dementia, and pronounced weight loss (3). Given that most of these negative prognostic features either improved or stabilized in our patient, it is reasonable to anticipate an ongoing TRKD-induced survival benefit. Alternative explanations for some of the improvements include a practice effect and a placebo effect. Although the former is possible, this seems unlikely given the lengthy 6-month time intervals between assessments. Since it is not possible to blind patients to metabolic strategies, a placebo effect may have partially contributed to the improvements.

In conclusion, this case study represents the first documented occurrence of a patient with ALS managed with either a fasting or ketogenic diet protocol, co-administered as a TRKD. We measured improved or stabilized ALS-related function, forced expiratory volume, forced vital capacity, swallowing impairment, neurocognitive status, depression, stress levels, and quality of life. Measurable declines were restricted to physical function, maximal inspiratory pressure, and maximal expiratory pressure. Our patient remains functionally independent and dedicated to his TRKD. Despite its limitations, this case study is encouraging and serves as a proof-of-concept for further studies involving metabolic strategies in ALS.

## Data availability statement

The original contributions presented in the study are included in the article/supplementary material, further inquiries can be directed to the corresponding author.

## Ethics statement

Ethical approval was not required for this study involving a human participant because local and institutional review boards do not require ethics approval for case reports/studies, so long as written informed consent is obtained. The study was conducted in accordance with the local legislation and institutional requirements. The participant provided written informed consent to participate in this study. Written informed consent was obtained from the individual for the publication of any potentially identifiable images or data included in this article.

## Author contributions

MP: Conceptualization, Formal analysis, Methodology, Supervision, Writing—original draft, Writing—review & editing. SJ: Data curation, Writing—review & editing. PS: Data curation, Writing—review & editing. DC: Data curation, Writing—review & editing. DM: Data curation, Writing—review & editing. RD: Data curation, Formal analysis, Methodology, Writing—review & editing.
